# Development of Value-Added Butter by Incorporating Whey Protein Hydrolysate-Encapsulated Probiotics

**DOI:** 10.3390/microorganisms11051139

**Published:** 2023-04-27

**Authors:** Kritika Gaba, Sanjeev Anand, Athira Syamala

**Affiliations:** 1 Midwest Dairy Foods Research Center, South Dakota State University, Brookings, SD 57007, USA; 2 Dairy and Food Science Department, South Dakota State University, Brookings, SD 57007, USA

**Keywords:** butter, probiotics, whey proteins

## Abstract

The probiotic foods market is growing exponentially; however, probiotics’ survivability and interaction with product attributes pose major challenges. A previous study of our lab developed a spray-dried encapsulant utilizing whey protein hydrolysate-maltodextrin and probiotics with high viable counts and enhanced bioactive properties. Viscous products such as butter could be suitable carriers for such encapsulated probiotics. The objective of the current study was to standardize this encapsulant in salted and unsalted butter, followed by storage stability studies at 4 °C. Butter was prepared at a lab-scale level, and the encapsulant was added at 0.1% and 1%, followed by physiochemical and microbiological characterization. Analyses were conducted in triplicates, and means were differentiated (*p* < 0.05). The viability of probiotic bacteria and the physicochemical characteristics of the butter samples with 1% encapsulant were significantly higher as compared to 0.1%. Furthermore, the 1% encapsulated probiotics butter variant showed a relatively higher stability of probiotics ratio (LA5 and BB12) than the control with unencapsulated probiotics during storage conditions. Although the acid values increased along with a mixed trend of hardness, the difference was insignificant. This study thus provided a proof of concept for incorporating encapsulated probiotics in salted and unsalted butter samples.

## 1. Introduction

With rising health awareness, the demand for functional foods is increasing. The consumption of dairy products has increased in recent years. This creates an opportunity for the dairy industry to incorporate bioactive ingredients like probiotics and whey proteins to increase the overall nutritional quality and functionality of dairy products [1]. The probiotics market is expected to increase exponentially at a Compound Annual Growth Rate (CAGR) of 6.81% from 2021–2028 globally and 12% from 2020–2025 in the United States alone [2,3]. However, the major concern with the incorporation of probiotics is their survivability during processing and under storage conditions. Previous studies have suggested a loss of about 2 log_10_CFU/g during digestion [4]. As per the International Dairy Federation, 10^6^–10^7^ log_10_CFU/mL is the minimum probiotics concentration to exert its claimed effects [5]. Many previous studies have attempted to incorporate probiotic organisms into dairy matrices directly. However, this resulted in interaction with the product, further impacting its organoleptic properties and a decline in probiotics viability over the storage period [6,7,8]. Out of all the strategies, encapsulation is perhaps the most effective technique for protecting the probiotics and retaining their bioactivity and viability [1,9,10]. Following this approach, a study of our lab successfully developed a spray-dried conjugated whey protein hydrolysate-maltodextrin (WPH-MD) probiotic encapsulant using the common probiotic strains *Bifidobacterium animalis* ssp. *lactis* ATCC27536 (BB12) and *Lactobacillus acidophilus* ATCC4356 (LA5) with 8.98 ± 0.02 log_10_CFU/g viable probiotic counts. The probiotic encapsulant also showed enhanced bioactive properties, like anti-hypertensive, antioxidant, anti-microbial, and storage stability for 16 weeks under refrigerated conditions (4 °C) [11].

The use of probiotics in dairy fermented foods and beverages like yogurt, Yakult, and cheese has been widely exploited. However, using non-fermented matrices like butter for incorporating encapsulated probiotics has yet to be extensively reported. Some studies have reported butter to be a potential carrier of LAB-based probiotic strains; however, this alters the product attributes [12]. Another study utilized fermented cream for butter production with LAB strain *L. helveticus* that showed 6 logs CFU/g with alterations in fatty acid composition [13]. Butter is an intricate water-in-oil emulsion with a prevalence of saturated fatty acids including lauric acid, myristic acid, palmitic acid, and fat-soluble vitamins such as Vitamin-E, carotenoids, and retinol [1,12,14]. It contains crystallized fat in which milk fat globules, partially coalesced milk fat globules, and water droplets are dispersed in a continuous phase [15]. Although fatty acids are a significant macronutrient in human diets, their high percentage (80%) in the matrix limits their consumption and acceptability by a section of consumers. Incorporating bioactive ingredients could thus improve their acceptability and nutritional value [2]. We hypothesized that butter could serve as a potential carrier of encapsulated probiotics with least interference with the product properties. Hence, the first objective of this study was to standardize the WPH-MD probiotic encapsulant in salted and unsalted butter matrix by incorporating it at 0.1% and 1%, followed by physicochemical characterization. The second objective of this study focused on storage stability studies under refrigerated conditions (4 °C) for 14 days.

## 2. Materials and Methods

### 2.1. Preparation of Spray-Dried Conjugated WPH-MD Probiotic Encapsulant

#### 2.1.1. Cell Harvesting of BB12 and LA5 for the Preparation of Concentrated Bacterial Cell Suspensions

The commercial strains BB12 and LA5 were obtained from ATCC. Both probiotic strains *Bifidobacterium animalis* ssp. *lactis* ATCC 27536 (BB12) and *Lactobacillus acidophilus* ATCC 4356 (LA5) were cultured in MRS (De Man, Rogosa, and Sharpe) broth (BD Difco^TM^, Fisher Scientific, Waltham, MA, USA) with the incubation conditions of 37 ℃, 72 h in an anaerobic jar using gaspak sachets (BD^TM^ Fisher Scientific, USA). After the activation and three sub-culturing steps, the cells were collected using centrifugation (1000× *g* for 15 min at 4 °C). The supernatant was removed, followed by 2–3 washings with the isotonic PBS solution (pH 7.2) to get the bacterial cells of BB12 and LA5. The cell concentration was further optimized at 0.8–0.9 OD, 600 nm using a spectrophotometer (Spectronic 200, ThermoFisher Scientific, Madison, WI, USA) to spike the conjugated solution at a concentration of 9–10 logs CFU/mL. The cell suspensions of BB12 and LA5 were held at 4 °C for inoculation in the conjugated whey protein hydrolysate solution.

#### 2.1.2. Preparation of Conjugated WPH-MD Probiotic Solution for Spray-Drying Process

The conjugated WPH-MD solution was prepared according to the protocol described by [11]. Briefly, the WPH10 and maltodextrin (DE10) (Sigma Aldrich, St. Louis, MO, USA; Fisher Scientific, Waltham, MA, USA) were mixed at a concentration of 5% *w*/*v* in two 1 L sterile bottles of distilled water at room temperature. The mixture was then solubilized at 25 °C for 2 h using magnetic stirrers (Corning, model number PC-420 D, Abington, MA, USA). The mixture’s pH (accumet basic AB 15 pH meter, Fisher Scientific, USA) was then adjusted to 8.2 using 0.5 N KOH. Next, the solution was hydrated at 4 °C for 18 h and heated at 90 °C for 24 h. The pH was again adjusted at 8.2 after the hydration step and the pH was recorded at 0, 3, 5, and 8 h intervals of heating. The conjugated solution was immediately cooled in iced water after heating. Then, the solution was inoculated with the probiotic (BB12 and LA5) cell suspensions. Both the cultures were spiked in a ratio of 1:1. The conjugated whey protein probiotic solution was then refrigerated at 4 °C until spray-drying.

#### 2.1.3. Spray-Drying of the Conjugated WPH-MD Probiotic Solution for the Probiotic Encapsulant Powder Preparation

The conjugated whey protein probiotic solution was spray-dried using a NIRO spray dryer in Davis Dairy Plant, SDSU. Spray drying of the conjugated whey protein probiotic solution was carried out as per the protocol described by [11]. The conjugated probiotic solution was fed into the system using a feeding pump after the conditions were stabilized at a pump flow rate of 110 mL/min and pressure of 379.21 kPa. The inlet and outlet temperatures were maintained at 200 °C and 90 ± 5 °C, respectively. The spray-dried whey protein–probiotic powder was collected from the cyclone collector in an airtight bag and stored at refrigerated conditions of 4 °C for further use.

### 2.2. Preparation of Value-Added Salted and Unsalted Butter Variants by Adding Encapsulated Probiotics

#### 2.2.1. Butter Sample Preparation under Laboratory Conditions

Raw cream was sourced from Davis Dairy Plant, SDSU with a fat content of 38%. It was divided into 200 g portions to prepare different variants of probiotic butter. 200 g of raw cream was subjected to batch pasteurization using a shaking water bath (BS-06 Lab Companion, Billerica, MA, USA) at 66 °C for 30 min as per PMO standards. The pasteurized cream was then tempered to 10 °C for the butter churning process. The cream was churned manually until the butter grains were separated from the buttermilk. Next, 2–3 washings were performed with distilled cold water [13].

#### 2.2.2. Incorporation of WPH-MD Probiotic Encapsulant in the Butter Grains to Develop Probiotic Butter Variants

Immediately after forming butter grains, the butter was spiked with probiotic encapsulant at two levels, 0.1%, and 1% *w*/*w*. The butter grains were homogenously mixed with the probiotic powder. For the salt-encapsulated probiotic butter variant, the salt was added at a concentration of 1.5% *w*/*w*. The probiotic butter was then kept in a sterile container at refrigerated conditions (4 °C) for further analysis.

### 2.3. Physicochemical Characterization of 0.1% and 1% Salted and Unsalted Encapsulated Probiotic Butter Variants

#### 2.3.1. Experimental Design

The WPH-MD probiotic encapsulant was incorporated at 0.1% and 1%, followed by their characterization. Furthermore, each concentration had salted (salt concentration of 1.5% *w*/*v*) and unsalted butter variants. Three independent trials were conducted. Each trial was performed in triplicate. One-way and two-way ANOVA were applied to differentiate between mean values. The analysis was done using MS Excel and R-studio version 2022.02.3 (Build 492).

#### 2.3.2. Enumeration of the Viable Probiotic Counts

The viable probiotic counts of salted and unsalted butter variants with the incorporation of WPH-MD probiotic encapsulant were enumerated by taking 1 g of sample tempered at room temperature in 9 mL of PBS. The tube was then kept in a water bath at 50 °C for 1–2 min to sufficiently mix the PBS with the emulsion. Then, serial dilutions were prepared using a PBS solution. 0.05% L-cysteine was added for the BB12 strain whereas LA5 was directly plated with MRS agar using the pour plate technique. The plates were incubated anaerobically at 37 °C for 72 h using a gaspak system. From the MRS (LA5) and MRS with L-cysteine plates (BB12), representative colonies were isolated and characterized through matrix-assisted laser desorption ionization-time of flight (MALDI-TOF). Three trials were conducted in triplicates.

#### 2.3.3. Physicochemical Analysis to Assess the Influence of the WPH-MD Probiotic Encapsulant on the Butter Properties

##### Effect of WPH-MD Probiotic Encapsulant on the Rheological Properties of Butter Variants

The frequency sweep measurements were taken for all the butter variants, 0.1% and 1% including control. The rheological analysis was performed using Anton Par Rheometer (MSR 92) with the following conditions: 40 mm parallel plate system at a frequency range of 0.1–100 Hz, shear stress of 10 Pa at a temperature of 25 °C. The samples were first equilibrated to room temperature [13]. The rheology measurements of loss and storage modulus and graphs for G′ and G″ were obtained in triplicates for each formulation.

##### Effect of WPH-MD Probiotic Encapsulant on the Textural Properties of Butter Variants

Textural properties of probiotic butter variants were analyzed using TA-XT plus texture analyzer (TA-XT plus, 6 Patton Drive, Hamilton, MA, USA). 0.1% and 1% butter variants along with the control sample were equilibrated at room temperature conditions followed by transferring to rectangular metal molds. These prepared rectangular metal molds with butter samples were stored at 4 °C for one day before the analysis. The sample dimensions were modified to 20 mm × 20 mm using a vernier caliper and wire cutter. Then, the textural analysis was carried out using the following conditions: cylindrical probe of 20 mm diameter, compression speed of 20 mm min^−1^ using uniaxial two-cycle compression [16]. The force–time curves were obtained for all the butter variants, and the parameters such as hardness (the maximum force required for the first compression cycle) and adhesiveness (the negative force from the first cycle) were interpreted [17].

### 2.4. Interaction of WPH-MD Probiotic Encapsulant with the Butter Matrix through Confocal Laser Scanning Microscopy

The interaction of the spray-dried WPH-probiotic encapsulant with butter matrix was evaluated using confocal laser scanning microscopy. Nile red (NR) and Fluorescein isothiocyanate (FITC) were the fluorescent staining agents for liquid fats and proteins, respectively. NR and FITC were dissolved in ethanol to a final concentration of 0.01%. After the churning procedure, the butter grains were mixed with 1 mL each of NR and FITC staining dyes. The samples were transferred to the pre-cooled slide. The solvents were made it possible to evaporate for five minutes, followed by covering the samples with a cover slip. The samples were then stored at refrigerated conditions for one hour before the microstructural analysis. The microstructural images were obtained at 40×, and 60× magnification with an Ar laser, and the fluorescent light emitted by NR and FITC was detected at 595–648 nm and 500–536 nm, respectively. The slides were adjusted in the white light, and live images were obtained with the Ar fluorescence laser. Images were captured using fine and coarse focus [15].

### 2.5. Storage Studies of Salted and Unsalted WPH-MD Probiotic Butter Samples at Refrigerated Conditions 4 °C

#### 2.5.1. Experimental Design

For the storage stability studies, 1% butter variants were prepared. Two types of variants were prepared, i.e., unsalted and salted (salt concentration of 1.5% *w*/*v*). For microbiological analysis, the controls were prepared with unencapsulated or free probiotic strains of BB12 and LA5. For physicochemical analysis including, controls were prepared without the incorporation of WPH-MD probiotic encapsulant. Both the salted and unsalted variants were analyzed at frequencies of 0, 7, and 14 days. The storage stability studies were conducted in triplicates.

#### 2.5.2. Probiotics Viability of Butter Samples during Storage

The viable probiotic counts were enumerated using the protocol described in Section 2.3.2. Analysis was conducted at frequencies of 0, 7, and 14 days for both the salted and unsalted butter samples.

#### 2.5.3. Impact of WPH-MD Probiotic Encapsulant on the Acid Value of Fatty Acids Present in Butter during Storage

A 50 g butter sample was melted in the oven for 2–3 h at 60 °C until the water and curd were separated. Melted butter was filtered through the Whatman-4 filter paper to get clear fat. Then, 5 g of well-mixed test proportion was mixed with 50 mL neutral ethyl alcohol in an Erlenmeyer flask, and 0.1 mL phenolphthalein solution was added. The mixture was brought to a boil in a water bath. Furthermore, the boiling mixture was titrated with 0.1 N alcoholic KOH until permanent faint pink appeared and persisted for ≥10 s. The acid value was obtained using the below formula:

Acid Value=56.1×N×V/W

where

*N*—Normality of KOH
*V*—Volume of KOH
*W*—Weight of the sample

#### 2.5.4. Impact of WPH-MD Probiotic Encapsulant on the Textural Properties (Hardness) of Butter during Storage

Analysis was conducted as per the protocol used to characterize the 0.1% and 1% butter variants. The salted and unsalted butter samples were procured during the storage frequencies of 0, 7, and 14 days during storage.

### 2.6. Statistical Analysis

Three independent trials were conducted. Each trial was performed in triplicate. Means were compared using one-way and two-way analysis of variance (ANOVA) using MS Excel and R-studio version 2022.02.3 (Build 492). Differences were considered significant at *p* < 0.05.

## 3. Results and Discussion

### 3.1. Viable Probiotic Counts in Salted and Unsalted Butter Variants with the Incorporation of WPH-MD Probiotic Encapsulant

Butter is a water-in-oil emulsion of ruptured, partially, and fully coalesced fat globules dispersed as a continuous phase. The oil entrapped inside the fat globules oozes out during churning and congregates to form a fat crystal network. Butter also contains an aqueous phase in which water droplets are present in a continuous phase [18]. The crystallized and viscous fat microstructure could potentially incorporate bioactive materials like probiotics. Encapsulant material would further provide a barrier between the probiotic organisms and the product matrix. The conjugated WPH-MD probiotic encapsulant was incorporated at two levels in the butter matrix, 0.1%, and 1%, for both the salted and unsalted variants.

As shown in Table 1, whipped cream with 1% WPH-MD encapsulated probiotics showed significantly higher viable probiotic counts for both the probiotic organisms, LA5 and BB12, compared to the 0.1% WPH-MD encapsulated probiotics. The 1% variants were preferred, as they demonstrated higher viability of about 5 logs CFU/g compared to the 0.1% variants with over 4 logs CFU/g. Based on these observations, butter was found to be a potential carrier for encapsulated probiotics. This could also be attributed to the presence of fatty acids and protein constituents that are known for their buffering capabilities and protection from external environmental conditions, as stated in some of the previous literature [19,20,21,22].

Previous studies have reported comparable results with different experimental conditions and food matrices. A study observed the survivability of probiotic strains as adjunct cultures along with the starter culture in the butter matrix at 4 °C for 60 days. Although it was a fermented product, comparable counts of *L. acidophilus* ATCC 4356 and *B. bifidum* ATCC 29521, up to 6 log_10_CFU/g were reported at the initial stage (second day) of storage time [12].

Another study reviewed the viability of encapsulated *L. acidophilus* and *B. bifidum* in a butter matrix using ion extrusion and gelation encapsulation techniques. In this study, the viability was monitored during refrigerated storage (at 5 °C). The encapsulant was added at higher concentrations (3, 5, and 10%). Although higher concentrations might have helped to retain higher probiotic counts, the initial probiotics inoculation data and the effect of the encapsulant on the butter’s physicochemical properties were not reported [4].

Both salted and unsalted butter variants retained comparable probiotic counts in both, 0.1% and 1% additions of the encapsulant. This further demonstrated the consistency of our study’s encapsulant inoculation in the butter matrix.

**Figure 1:**
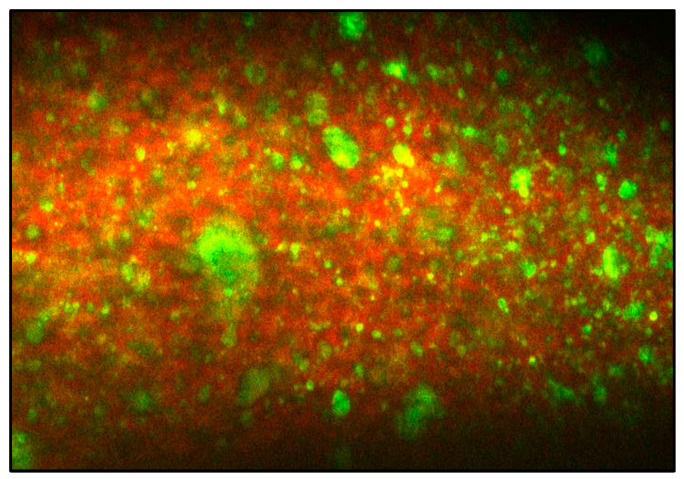
Confocal Laser Scanning micro image of WPH-MD probiotic encapsulant in butter matrix. Nile Red Fluorescence (red) represents fat phase and Fluorescein Isothiocyanate (green) represents protein bodies.

While standardizing the incorporation of WPH-MD encapsulant in butter matrix, confocal laser scanning image showed a distribution pattern of the fat globules (red color, stained with Nile Red dye) and protein bodies including encapsulant (green color, stained with Fluorescein Isothiocyanate) as shown in Figure 1.

Overall, the results depicted the suitability of the butter matrix for incorporating encapsulated probiotics. The 1% variant was preferred due to higher probiotics viability as compared to the 0.1% variant. Based on these findings, storage stability studies were performed with 1% WPH-MD probiotic butter to further observe the survivability of probiotics (LA5 and BB12) during refrigerated storage.

### 3.2. Effect of WPH-MD Probiotic Encapsulant on the Rheological Properties of Butter Variants

Milk products like butter undergo various structural changes during mechanical treatments such as the heating of cream, homogenization, and phase inversion during the churning process. These structural changes affect the chemical composition like nucleation properties and microstructure of the butter matrix. Such chemical changes expose the fat matrix to other components like proteins affecting butter’s rheology and textural properties [15]. Rheological measurements were taken to evaluate the effect of WPH-MD probiotic encapsulant containing whey protein hydrolysates and probiotics on the structure of butter.

**Figure 2:**
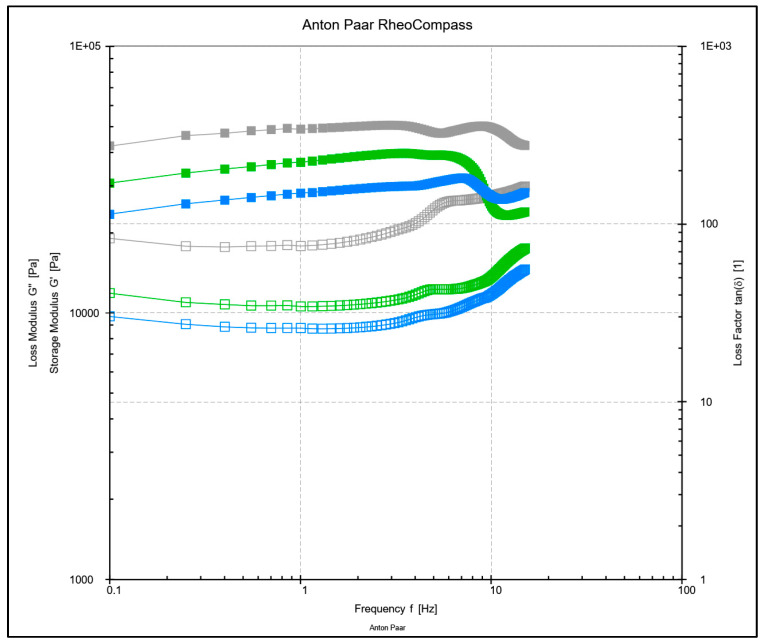
Frequency sweep measurements (storage and loss modulus) using a parallel plate system of 1% WPH-MD probiotic, 0.1% WPH-MD probiotic and control unsalted butter samples. The solid lines represent the trend lines for storage modulus (G′) and non-solid represents the trend lines for loss modulus (G″). The grey colored trend line represents the 1% WPH-MD probiotic unsalted butter variant, green represents 0.1% WPH-MD probiotic butter variant, and blue represents control with no WPH-MD probiotic encapsulant addition.

Unsalted butter variants showed the highest rheological properties, including storage and loss modulus as shown in Figure 2. It was found that storage (G′) and loss modulus (G″) increased with the increase in frequency indicating a solid-like behavior at ambient temperatures. G′ was greater than G″ in all the butter formulations including 1% WPH-MD probiotic unsalted, 0.1% WPH-MD probiotic unsalted, and control with no encapsulant reflecting the viscoelastic behavior. This could be due to the deposition of proteins on the fat globules in the continuous phase indicating a strong and stabilized viscoelastic network with enhanced storage and loss modulus.

A previous study reported also observed G′ > G″ and both storage and loss modulus increased with the increase in frequency in a different butter variant (low-calorie pistachio). This was further linked to Deborah’s number (De), which relates the solid-like behavior of the materials with an increase in frequency [17]. These results were in parallel with the observations of another study which studied the effect of emulsifiers like whey protein isolates and whole milk powder on the rheological properties of butter through frequency sweep measurements. This study indicated a linear correlation between the addition of whey proteins on the hardness and stiffness of butter compared to the control [20].

Another study demonstrated comparable findings with other emulsions. The study showed that the elastic modulus was higher than the loss modulus showing a solid gel behavior of emulsions prepared with butter and corn oil. The other influencing parameter can be the presence of globular water in the continuous phase. The presence of the aqueous phase in the continuous phase increases the rigidity and viscosity of the butter matrix [23].

**Figure 3:**
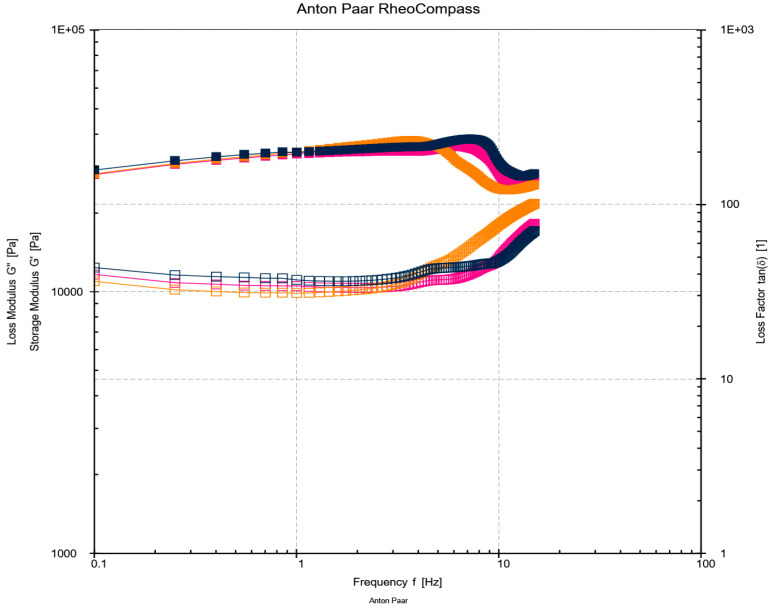
Frequency sweep measurements (storage and loss modulus) using a parallel plate system of 1% WPH-MD probiotic, 0.1% WPH-MD probiotic and control salted butter samples. The solid lines represent the trend lines for storage modulus (G′) and non-solid represents the trend lines for loss modulus (G″). The pink colored trend line represents the 1% WPH-MD probiotic unsalted butter variant, yellow represents 0.1% WPH-MD probiotic butter variant, and black represents control with no WPH-MD probiotic encapsulant addition.

Figure 3 depicts the rheological behavior of salted butter variants. It showed an insignificant trend as the presence of mono-valent salt ions including Na+ ions at a lower concentration, decreases the modulus properties due to the charge-shielding effect resulting in fewer interactions. On the other hand, an increased concentration of mono-valent ions forms linkages with the water molecules and provides strong structural interactions [24,25]. In our study, salt concentration was kept constant at a lower level of 1.5% *w*/*w*. Previous studies have also reported results of decreasing or no change in moduli with the presence of chloride ions.

One of the studies indicated the elastic response, that is, G′ > G″ for the probiotic and aromatic butter variants. In contrast, a viscous response, G″ > G′, was recorded for sweet and kefir butter variants. These results revealed a solid-like behavior further indicating recoverable deformations [25]. The 0–200 mM concentration of chloride ions does not alter the moduli drastically compared to the addition of Ca^2+^, causing cross-linkages with carboxyl functional groups resulting in modified structural interactions [26]. Overall, adding the WPH-MD probiotic encapsulant at a moderate concentration did not impact the rheology of the butter matrix.

### 3.3. Effect of WPH-MD Probiotic Encapsulant on the Textural Properties of Butter Variants

The texture is one of the four major factors in developing a food product, reflecting the overall consumer acceptability [20]. The textural property of butter is linked to its multiphase conversion that occurs during the churning process. The mechanical force during the churning process ruptures the milk fat globule membrane present on the fat globules in the emulsion phase and brings out the liquid fat from the globules to the continuous phase. The coalesced fat crystals aggregate after the butter grains have been separated from the buttermilk. Van der Waals forces hold these crystals of coalesced, partially coalesced, and ruptured fat, establishing a 3-dimensional fat crystal network governing butter’s further textural and structural characteristics [27]. Additionally, water droplets present in the continuous phase of butter play a significant role in determining butter’s firmness and textural parameters. The aqueous phase contributes 15–20% of the butter’s mass and contributes to the butter’s viscosity and rigidity.

**Figure 4:**
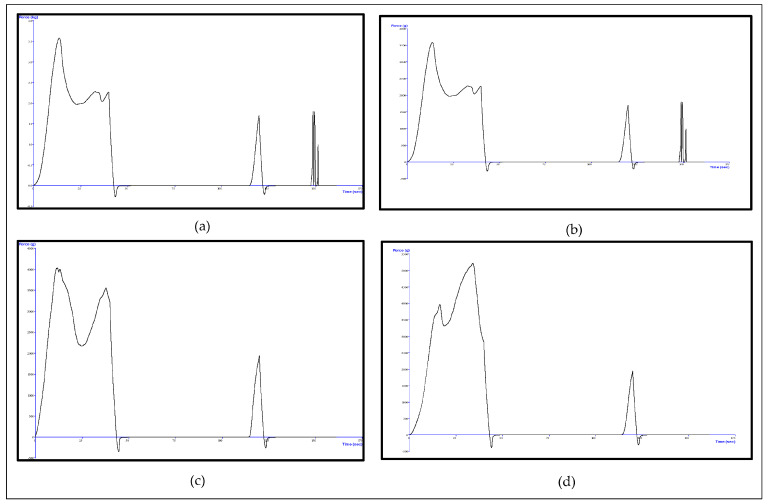
Textural Profile Analysis of 1% WPH-MD probiotic butter variants. (**a**) TPA of 1% WPH-MD probiotic unsalted butter; (**b**) TPA of unsalted control; (**c**) TPA of 1% WPH-MD probiotic salted butter; (**d**) TPA of salted control.

As shown in Figure 4, the 1% probiotic butter variants showed the lowest peaks for hardness, i.e., the force required for the first compression cycle, whereas control had the highest peak for hardness. The presence of water droplets in the continuous phase of butter increased the rigidity and hardness. This hardness further requires more energy to reduce the surface tension for the emulsion. The control did not contain an emulsifier; therefore, it required more energy or force for the compression and gave the highest peak for the hardness. Whey proteins are known for their surface-active and amphiphilic properties that reduce the interfacial tension between the w/o surface. Hence, with the incorporation of whey proteins, the hardness was found to be reduced.

As for the adhesiveness parameter, 1% probiotic butter samples required a force i.e., the negative area under the force which was less than the control. This could be due to the established 3-dimensional network of fat crystals in butter production. The increase in viscoelastic characteristics, as shown through rheological parameters with incorporating the probiotic encapsulant, strengthens the network. Therefore, the incorporation of the whey proteins through the encapsulant had a positive impact on the textural properties of butter. This further showed that the conjugated whey protein hydrolysate probiotic encapsulant did not interfere with the textural properties of butter.

### 3.4. Viability of Encapsulated Probiotics in Salted and Unsalted Butter Variants during Refrigerated Storage Conditions:

From the above microbiological and physicochemical characterization, 1% salted and unsalted butter variants exhibited significantly higher viable probiotic counts (LA5 on MRS and BB12 on MRS, L-cysteine) and product attributes including rheological and textural properties compared to the 0.1% salted and unsalted butter variants. Based on this, storage studies were conducted with 1% salted and unsalted butter variants at refrigerated conditions (4 °C). Both variants were compared to their controls containing free or unencapsulated probiotic strains.

**Figure 5:**
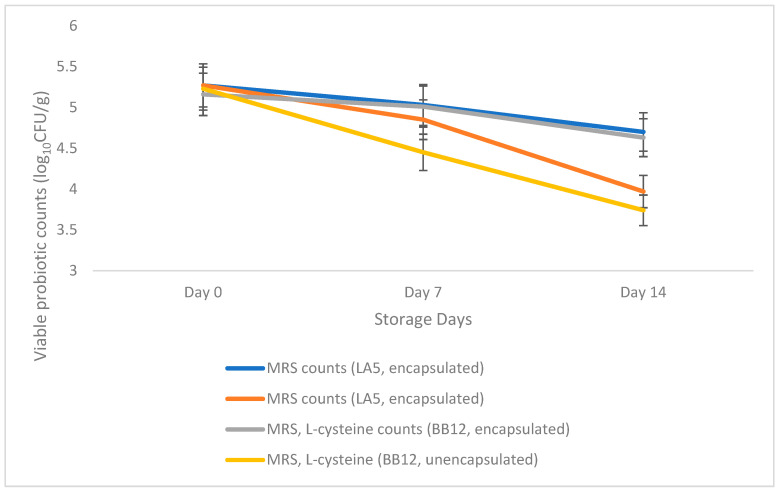
Viable probiotic counts of (LA5 on MRS and BB12 on MRS, L-cysteine) unsalted butter samples during storage (4 °C).

The encapsulated probiotics (LA5 and BB12) in unsalted and salted butter variants showed significantly higher viable probiotic counts than the control containing unencapsulated probiotics. As shown in Figure 5, the trend line for encapsulated probiotics did not show a substantial drop in viability. On the other hand, control showed an overall decrease in probiotic viability during the refrigerated storage. The encapsulated and unencapsulated butter variants were around 5 and 4 logs CFU/g, respectively at the end of storage time (14th day).

A previous study also reported a decline in the viability of *L. acidophilus* and *B. bifidum* when added as free cells during storage at 5 °C in the butter matrix [4]. Another study showed around 6 logs CFU/g viability of *L. casei* up to 60 days of the storage period with the addition of bixin antioxidant effect that might have helped to improve the overall shelf stability of butter [1].

Other studies showed the survival of probiotic strains in different product matrices. A study showed the survival of *L. rhamnosus* GG populations in full-fat and reduced-fat peanut butter stored at different temperatures (4, 25, 37 °C). Factors such as probiotic culture inoculation at 10^7^ CFU/g and low moisture content in peanut butter might have helped to retain more than 6 logs at 4 °C for 48 weeks. Overall, it showed the viability of probiotics ranging from 5.97 logs CFU/g to 4.13 logs CFU/g during the 48 weeks of the storage period [19].

**Figure 6:**
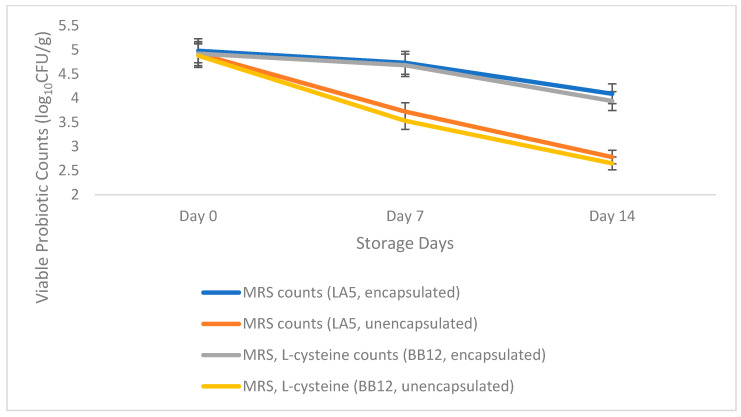
Viable probiotic counts of (LA5 on MRS and BB12 on MRS, L-cysteine) salted butter samples during storage (4 °C).

The encapsulation provided further protection to maintain the ratio of LA5 and BB12 at 1:1 during the refrigerated storage conditions. As shown in Figure 5 and Figure 6, the encapsulated trend lines for LA5 and BB12 did not show a difference in their viability during storage. However, unencapsulated LA5 and BB12 showed a substantial difference in their trend lines. This could be attributed to the reason that the free probiotic organisms might have been exposed to external environmental conditions resulting in a decline in their viability.

In addition, LA5 showed relatively higher stability as compared to BB12 in the unencapsulated probiotics butter variant. Previous literatures have reported that lactobacilli are usually more resistant to environmental conditions as compared to Bifidobacteria. Additionally, some of the metabolic products released by lactic acid bacteria could inhibit the growth of Bifidobacteria [4].

It was also observed that the salted variants showed relatively lower viable probiotic counts as compared to the unsalted variants. Although no related studies were found, the presence of salt might have impacted the overall viability of probiotic organisms. Some of the previous literature has mentioned that the LAB, in general, is resistant to bile salt and hence, salt concentrations [28]. However, it also depends on various factors including salt concentration, probiotic strains, bacterial population, and the release of bioactive substances during the storage time. This observation would need to be investigated further to establish the impact of salt, if any, on the viability of encapsulated probiotics.

Overall, adding the encapsulant provided reasonable stability by retaining the viability of the probiotics for both the organisms, LA5 and BB12, at a ratio of 1:1 as compared to the unencapsulated probiotics variants during storage. Therefore, this storage study utilized the butter matrix as a carrier for the encapsulated probiotics incorporation without affecting the product attributes.

### 3.5. Impact of Incorporation of WPH-MD Probiotic Encapsulant on the Acid Value of Salted and Unsalted Butter Variants during Refrigerated Storage Conditions

The acid value is one of the quality parameters of fat-rich products that evaluates lipolysis. It can be defined as the number of free acids in fat, measured in terms of the number of milligrams of potassium hydroxide needed to neutralize them. The acid content in the fat is given by the quantity of free fatty acids released from triacylglycerol during hydrolytic deterioration (rancidity). This alteration occurs under unsuitable conditions of treatment and preservation of fats. Butter is a fat-rich dairy product with high water content makes it more susceptible to hydrolysis leading to an unpleasant smell and taste.

**Figure 7:**
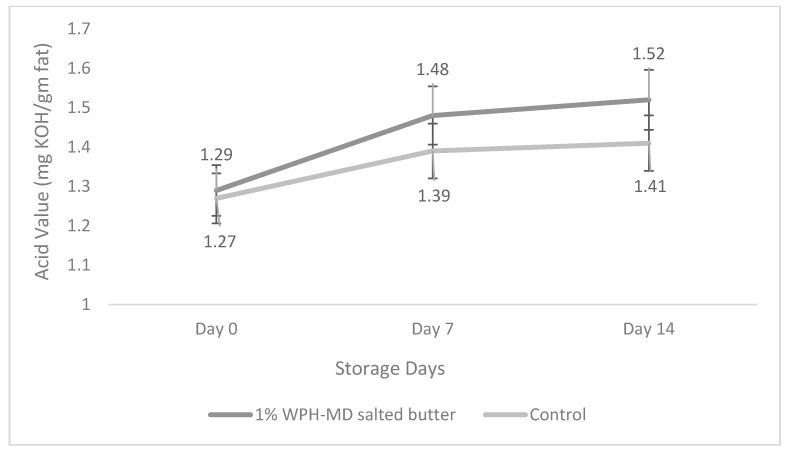
Acid value of salted WPH-MD probiotic butter variants during storage (4 °C).

For the salted WPH-MD probiotic butter samples, in both treated and control, there was an insignificant increase in the acid value from 0 to the 14th day of storage at refrigerated conditions, as shown in Figure 7. Although 1% WPH-MD probiotic salted butter samples showed higher acid values during the storage time than the control, there was no significant difference between the two during the storage. The marginal increase in acid values can be due to the disintegration of fatty acids during the storage due to the action of lactic acid bacteria, including BB12 and LA5, that might have resulted in the increase in acid values [13].

**Figure 8:**
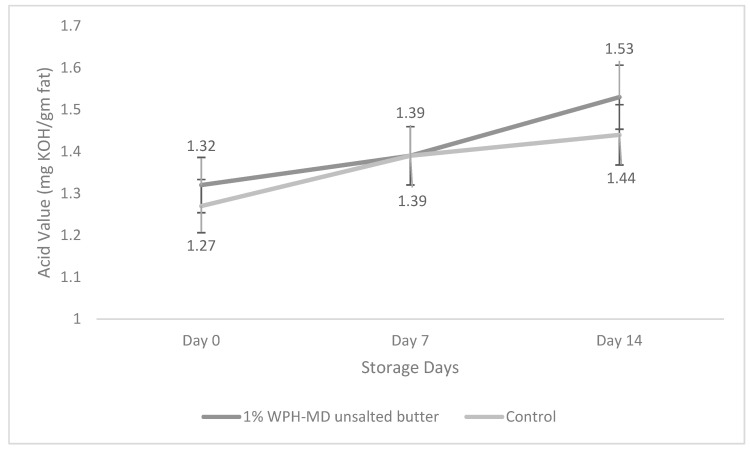
Acid value of unsalted WPH-MD probiotic butter samples during storage (4 °C).

As for the unsalted WPH-MD probiotic butter samples, similar trends were observed as shown in Figure 8. 1% WPH-MD probiotic unsalted butter sample and control did not show any significant difference in their acid values during the storage. Although control showed significantly higher acid values from 0–14 days, 1% WPH-MD probiotic unsalted butter did not show a drastic increase in their acid values during the storage. The marginally higher acid values of 1% WPH-MD probiotic butter samples could be attributed to the production of organic acids from probiotic organisms, LA5 and BB12. In addition, since unsalted butter samples showed higher viabilities than salted ones, unsalted showed higher acid values due to the higher concentration of BB12 and LA5.

Many previous studies have shown a significant increase in acid values with direct probiotics incorporation. A couple of studies reported increased acidity with adding LAB, including *L. helveticus* producing organic acids [6,13]. Another study observed a slow change in the acid value of butter samples from 0 to 8 weeks [29]. The results were further in agreement with another study that reported an increase in the acid values of butter [30]. From the above results, it can be interpreted that encapsulation protected the probiotic organisms that did not result in significant changes in the acid values of butter which further represents the oxidative quality of butter. Therefore, the addition of the WPH-MD probiotic encapsulant led to an inconsequential increase in acid value in both salted and unsalted butter samples as compared to control.

### 3.6. Impact of Incorporation of WPH-MD Probiotic Encapsulant on the Textural Properties of Salted and Unsalted Butter Variants during Refrigerated Storage Conditions

**Figure 9:**
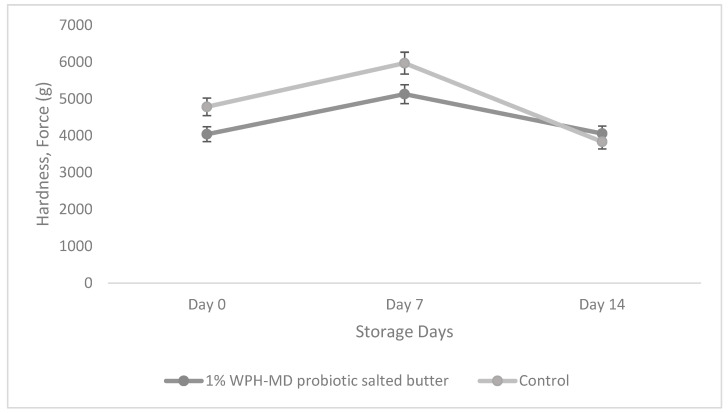
Hardness of salted WPH-MD probiotic butter samples during storage at refrigerated conditions (4 °C).

The textural properties of a complex matrix like that of butter depend on several factors including interaction and networking at the interfacial layer, fat globule size, and proportion of the solid/liquid fat fraction. The textural properties, mainly hardness, was evaluated for the salted and unsalted butter samples during the 14-day storage period. Hardness can be defined as the stress required to break the network of fat globules. The textural attributes of the butter samples were investigated through large deformation techniques, as presented in Figure 9.

According to the TPA results, 1% WPH-probiotic salted butter showed a decreasing hardness trend with the storage time compared to control salted butter. However, the difference was not significantly different. The hardness of both 1% and control butter variants increased until Day 7 of storage. However, it decreased after that until Day 14 of storage. Although it showed a mixed trend, the change in hardness was insignificant for both the individual (1% WPH-MD probiotic salted butter and control) samples during the storage time. The increase in the hardness trend in the first week of storage might indicate the strong network with the addition of the WPH-MD probiotic encapsulant. As the storage time increased, the hardness decreased. This might be due to the reason that the protein and the fat network were intervened. In this storage study, we only evaluated the hardness parameter of TPA; however, further studies might be required to study the other parameters of TPA, including adhesiveness, spreadability, and viscosity index.

**Figure 10:**
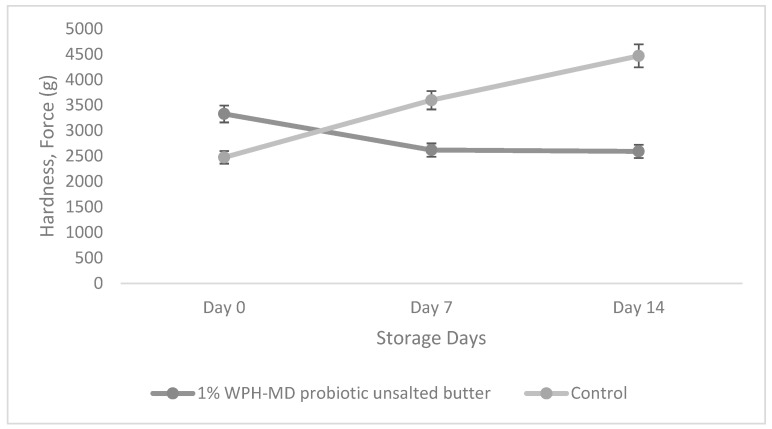
Hardness of unsalted WPH-MD probiotic butter variants during storage at refrigerated conditions (4 °C).

In unsalted samples, 1% WPH-MD probiotic unsalted butter showed insignificantly lower hardness values compared to the control as depicted in Figure 10. This could be due to the presence of high moisture content in unsalted butter variants. Although the unsalted samples also showed a mixed trend of decreasing and increasing hardness, the hardness values were insignificant for the individual samples and amid each other during the storage time. This might be due to microencapsulated particles’ small amount incorporation and size into the butter matrix.

Some of the previous studies have indicated a decrease in hardness parameters by adding different proteins. A study evaluated the impact of rice flour on the sensory and the TPA profile of butter cakes. The author has reported an improved TPA profile by adding rice flour containing amylose owing to the development of a rapid network [31]. Another study reported an increase in the firmness trend followed by a decrease in the hardness in the later storage stages owing to the weakening of the protein network due to fat presence [20]. Overall, it was observed that the incorporation of 1% WPH-MD probiotic formulation did not affect the hardness of butter significantly compared to the corresponding control.

## 4. Conclusions

Increasing the functionality and bioactivity of dairy products without interfering with the physicochemical properties is a growing research area. This study developed a value-added range of butter variants by incorporating a WPH-MD probiotic encapsulant at 0.1%, and 1%. The 1% butter variants showed significantly higher probiotic (LA5 and BB12) counts and physicochemical characteristics, including rheological parameters and TPA, compared to the 0.1% butter variants. Further storage studies with 1% butter variants showed acceptable probiotic (LA5 and BB12) counts of over 5 and 4 logs CFU/g in unsalted and salted butter variants, respectively. Encapsulation also helped maintain the 1:1 ratio of the organisms, LA5 and BB12, during storage conditions. On the other hand, the control showed higher tolerance of LA5 compared to BB12. Adding the WPH-MD probiotic encapsulant to the butter did not alter the physicochemical properties significantly. Hence, this study proved to utilize butter as a potential carrier of probiotics without affecting the product attributes. Further studies on extended storage stability of encapsulated probiotics in butter would be necessary for scaling up the process.

## Figures and Tables

**Table 1:** Viability (log_10_CFU/g) of encapsulated probiotics (LA5 and BB12) in 0.1% and 1% unsalted and salted butter variants.

Butter Samples	Viable Probiotic Counts on MRS Plate (LA5) (log_10_CFU/g)	Viable Probiotic Counts on MRS, L-Cysteine Plate (BB12) (log_10_CFU/g)
0.1% WPH-MD Encapsulated probiotic unsalted butter	4.31±0.84 ^aA^	4.28±0.47 ^aB^
1% WPH-MD Encapsulated probiotic unsalted Butter	5.18±0.32 ^bA^	5.16±0.53 ^bB^
0.1% WPH-MD Encapsulated probiotic salted Butter	4.29±0.27 ^cA^	4.27±0.94 ^cB^
1% WPH-MD Encapsulated probiotic salted Butter	4.98±1.04 ^dA^	4.92±0.50 ^dB^

Means±SE accompanied by the lowercase represents column comparison and uppercase represent row comparison using ANOVA analysis *(*p≤0.05).

## Data Availability

All data used to support the findings of this study are included within.
